# Translating Evidence for Low Back Pain Management into a Consumer-Focussed Resource for Use in Community Pharmacies: A Cluster-Randomised Controlled Trial

**DOI:** 10.1371/journal.pone.0071918

**Published:** 2013-08-20

**Authors:** Helen Slater, Andrew M. Briggs, Kim Watkins, Jason Chua, Anne J. Smith

**Affiliations:** 1 School of Physiotherapy, Curtin University, Perth, Western Australia, Australia; 2 Curtin Health Innovation Research Institute, Curtin University, Perth, Western Australia, Australia; 3 Department of Health, Government of Western Australia, Perth, Western Australia, Australia; 4 Arthritis and Osteoporosis Victoria, Melbourne, Victoria, Australia; 5 School of Medicine and Pharmacology, University of Western Australia, Perth, Western Australia, Australia; Iran University of Medical Sciences, Islamic Republic of Iran

## Abstract

**Background:**

This cluster-randomised controlled trial determined the effectiveness of an evidence-based, pamphlet intervention in improving low back pain (LBP)-related beliefs among pharmacy consumers.

**Methods:**

Thirty five community pharmacies were randomised to three groups: pamphlet+education intervention [n = 11]; pamphlet only intervention [n = 11]; control: usual care [n = 13]. Eligibility requirements for clusters included: community-based pharmacies and proprietor participation consent. Pharmacy consumers (N = 317) aged 18–65 years currently experiencing LBP participated. Intervention group allocation depended on the pharmacy attended. Individual-level outcomes were measured at pre-intervention (T0), at two (T1) and eight (T2) weeks post-intervention and included beliefs about LBP [Back Pain Beliefs Questionnaire (BBQ); Fear Avoidance Beliefs Questionnaire (FABQ)]. Secondary outcomes included pain severity, activity impairment and pamphlet perceived usefulness. Blinding to group allocation included primary investigators, outcome assessors and the statistician. Pharmacy staff and consumers were un-blinded.

**Results:**

Of 35 pharmacies recruited (317 consumers), no clusters were lost to follow-up. Follow-up was available for n = 24 at 2 weeks only; n = 38 at 8 weeks only; n = 148 at both time points, with n = 148+24+38 = 210 analysed (107 excluded: no follow up). Adjusting for baseline scores demonstrated no significant differences in beliefs (2 or at 8 weeks) between pamphlet (with or without education) versus control, or between ‘pamphlet with’ versus ‘without’ education. Work-related fear (FABQ) was significantly lower in consumers receiving pamphlet (with or without education) versus control (difference −2.3, 95%CI: −4.4 to −0.2). There was no significant difference between “pamphlet with” versus “pamphlet without” groups. Consumers receiving the “pamphlet with” reported greater perceived usefulness than consumers receiving the “pamphlet without” (difference 0.9 (95%CI: 0.0 to 1.8)).

**Conclusion:**

Community pharmacies provided a feasible primary care portal for implementing evidence-based information. The associated improvement in work-related LBP-beliefs for consumers receiving the pamphlet suggests this simple intervention may be a useful component of care.

**Trial Registration:**

ACTR.org.au ACTRN12611000053921

## Background

Low back pain (LBP), particularly persistent LBP, continues to present a complex and challenging problem for consumers, healthcare professionals, health delivery systems, and health policymakers [1]. The escalating costs of health services directed at arresting the health and economic burden associated with LBP syndromes are unsustainable [2], and the individual and societal burdens highlight the urgency to reconsider how management for consumers with LBP might be better undertaken within the context of primary care practice[3]–[5]. In particular, the role of the consumer in the co-operative management of their acute [6], [7] and persistent LBP [8] is fundamental to optimising recovery [9]. However, in order to actively participate in effective co-care, consumers need reliable, accessible, meaningful and understandable health information, particularly at the community level [5], [10]. Our study aimed to address this gap in knowledge about what constitutes feasible and effective primary care implementation of reliable consumer information regarding LBP.

One contemporary strategy to facilitate the uptake of evidence-informed LBP management by individual health professionals, and the effective translation of this information to consumers, is by using a community of practice framework[11]–[13] to promote intra- and inter-disciplinary continuing professional development and to encourage consistent best practice integrated care. In this context, community pharmacies can be seen as a key partner in a healthcare community of practice and are an obvious primary health information portal for consumers [14], particularly considering the high volume of analgesic usage and prescriptions associated with LBP management[15]–[18]. Community pharmacies may represent a potentially under-utilised health workforce for providing accessible and evidence-based information about LBP to consumers. A recent cluster randomised trial [19] demonstrated that a pharmacy-initiated multidisciplinary intervention was feasible and effective in delivering positive patient outcomes for people with osteoarthritis. However, currently there is a gap in understanding the role of primary care portals such as community pharmacies, in contributing to the management of chronic conditions such as LBP. The need is evident for accessible consumer resources promoting consistent, evidence-based messages which are adopted in primary care and which align with current recommendations for LBP management [20] and health policy [21], [22].

Using the same evidence-informed resource simultaneously throughout communities of practice and across the diversity of health disciplines may encourage the adoption of more positive LBP beliefs and behaviours, as demonstrated by Buchbinder et al. [23] in response to a successful population health strategy undertaken in Victoria, Australia. The provision of a single, credible resource providing evidence-informed messages, underpinned by health policy [5] and designed for use by both health professionals and consumers with LBP, may encourage the adoption of more consistent LBP management in primary care. In this context, an emerging body of evidence points to the benefits of education booklets and pamphlets for improving knowledge and outcomes for consumers with LBP[24]–[28]. A recent systematic review [26] determined that pamphlets based on a biopsychosocial model of pain rather than a biomedical model, were associated with improved consumer beliefs regarding physical activity, pain and consequences of LBP. Further, supporting information with verbal reinforcement from health professionals may improve outcomes to a greater extent [24], [26], provided that the information is not overly detailed [28].

As the use of a cluster randomised trial (C-RCT), minimises the likelihood of cross-group contamination [29] and furthermore is recommended as appropriate for evaluating community-based health promotion [19] or education initiatives [30], we employed the rigour of a C-RCT with the objectives of determining, at an individual level, the effectiveness of: (i) a consumer LBP pamphlet compared to usual pharmacy care in improving LBP-related beliefs among community pharmacy consumers with LBP; and (ii) delivering a pamphlet with and without additional verbal reinforcement of the pamphlet key messages by pharmacy staff. These objectives were achieved.

## Materials and Methods

The protocol for this trial and supporting CONSORT checklist are available as supporting information; see Checklist S1 and Protocol S1.

### Purpose

As a policy implementation initiative, the Western Australian Musculoskeletal Health Network (http://www.healthnetworks.health.wa.gov.au/network/musculoskeletal.cfm) tasked an expert, interdisciplinary working group to develop a pamphlet for use by consumers with LBP in a primary care setting in Western Australia (WA). (The pamphlet can be viewed at: http://www.healthnetworks.health.wa.gov.au/docs/2010_BackPain.pdf). The interdisciplinary group included the following stakeholder groups: health consumers; pharmacy; pain medicine speciality; rheumatology speciality; neurosurgery speciality; musculoskeletal physiotherapy; general medical practice; chiropractic; psychology and health policy. The pamphlet was developed explicitly on evidence-based LBP clinical guidelines, used consumer-oriented language and was developed within a biopsychosocial framework, consistent with key recommendations outlined in state health policy for the management of spinal pain: ‘The WA Spinal Pain Model of Care’ [5]. Since evidence suggests that outcomes for patients with LBP may be improved when written health information is reinforced by a health professional [24], [26], this pamphlet was designed with key messages that health professionals could quickly and easily reinforce to consumers. The pamphlet underwent a period of stakeholder consultation within the Network and with key professional and consumer organisations. The final copy of the resource was endorsed by the Australian Clinical Psychology Association, Australian and New Zealand College of Anaesthetists (Faculty of Pain Medicine), Australian Osteopathic Association, Australian Pain Society, Australian Physiotherapy Association, Australian Rheumatology Association (WA), Chiropractors’ Association of Australia, Health Consumers’ Council, Pharmaceutical Society of WA and the Royal Australian College of General Practitioners.

### Trial Design

**Figure 1:**
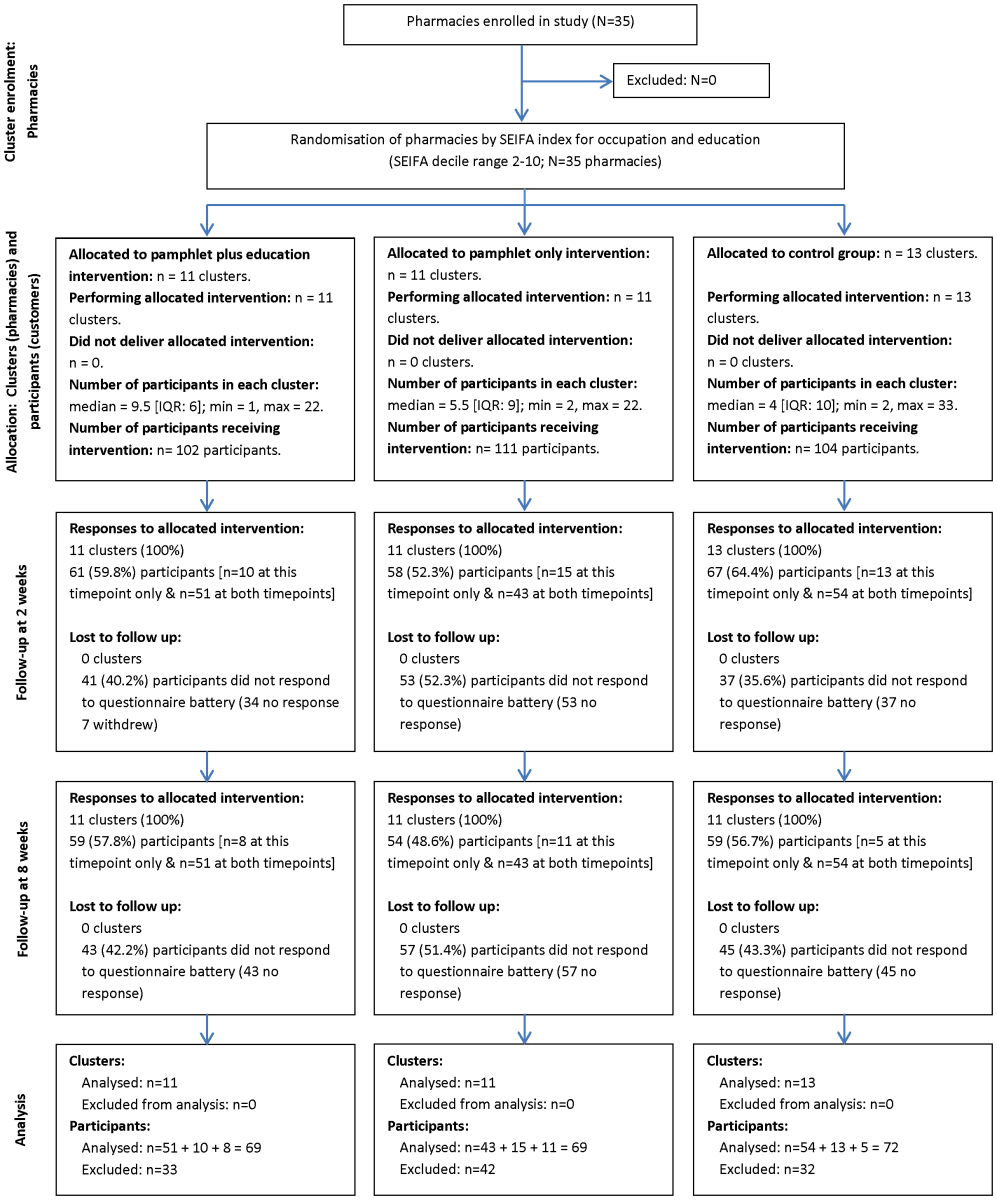
Flow diagram of progress of clusters and participants through phases of the cluster-randomised controlled trial. This study was undertaken in 35 community pharmacies in metropolitan Perth, WA. An index of education and occupation was assigned to each participating pharmacy based on the Australian Bureau of Statistics Socioeconomic Index for Area (SEIFA) [32]. The ascending distribution of indices across the pharmacies (range: 2–10) was divided into thirds, such that a low, medium and high SEIFA group was created. Pharmacies from within each SEIFA block were then randomised to one of three cluster groups: two intervention groups (pamphlet with education [n = 11]; pamphlet only [n = 11]; and a control (usual care) group [n = 13]) and within each cluster group, the SEIFA range was 2–10, representing an equal spread of education and occupation status across the study clusters. Recruitment occurred via three routes: (i) consumers approached the pharmacist with a prescription for analgesia related to LBP; or (ii) requested non-prescription medication for management of their LBP; or (iii) inquired about the study after seeing study posters within the pharmacy. Pharmacy consumers were then invited to participate in the study if they were currently experiencing LBP, were aged between 18–65 years, and could read and comprehend English.

In the initial planning phase, a randomised controlled trial (RCT) design was proposed for this project (see Protocol S1), however since it became evident that it was more feasible to randomise pharmacies, rather than pharmacy consumers to the intervention [19], [30], we subsequently employed a cluster randomised trial (C-RCT) design [29]. This design was employed prior to initiation of the trial and prior to any data collection. Therefore, while the C-RCT design we implemented was different from the RCT design initially detailed in our protocol, the execution of the trial and the analytic approach still adhered to the same study protocol. For this C-RCT, the community pharmacies were defined as the ‘clusters’, while the consumers were ‘participants’ in these clusters. Reporting of this trial is consistent with the CONSORT 2010 statement: extension to cluster randomised trials [31] and is supported by a cluster and participant flow diagram showing the progression of participants from group assignment to final analysis (Figure 1 and Checklist S1). Not all components of the recommended CONSORT flow diagram are reflected in Figure 1 owing to the specific features of this trial, as recommended by Campbell et al [31]. Primary outcomes were analysed at the individual level, adjusting for within-pharmacy correlation in outcome measures.

### Ethics Statement

The study was approved by the local institutional Curtin University Human Research Ethics Committee (HR 171/2010), complied with the Declaration of Helsinki and was registered with the Australian and New Zealand Clinical Trials Register (ACTRN12611000053921) and allocated a Universal Trial Number U1111-1119-0440**.** Written informed consent was obtained from all individual participants. The proprietors of all pharmacies also provided consent to participate as a cluster member, and this consent was provided prior to randomisation.

### Setting, Eligibility and Recruitment

This study was undertaken in 35 community pharmacies in metropolitan Perth, WA. Recruitment occurred between May 2011 and August 2011 (trial completion), this later date reflecting the exhaustion of recruitment resources and funding. Participating pharmacies were identified based on an expression of interest issued by the Pharmaceutical Society of WA (PSWA) (http://www.pswa.org.au). The inclusion criteria included: (i) the pharmacy proprietor agreed to the practice being involved; and (ii) qualified staff within the pharmacies willing to facilitate participant recruitment. The sole exclusion criterion (cluster level) was the pharmacy proprietor not agreeing to be involved in the study. A liaison community pharmacist (KW) discussed the eligibility, roles and responsibilities of participating pharmacies with those PSWA members who expressed an interest in the study. Pharmacies were remunerated for their involvement, at the rate of $AU10 for each participant recruited into the trial.

Consumer participants were recruited via three routes: (i) consumers approached the pharmacist with a prescription for analgesia related to LBP; or (ii) requested non-prescription medication for management of their LBP; or (iii) inquired about the study after seeing study posters displayed within the pharmacy. Pharmacy consumers were then invited to participate in the study if they met the following inclusion criteria: (i) were currently experiencing LBP; (ii) were aged between 18–65 years; and (iii) could read and comprehend English. Criteria to establish the nature (specific or non-specific) of the low back pain were not included, as the professional skills required to classify LBP were considered a barrier to implementation and beyond the scope of practice for community pharmacists. Further, such a classification approach, while ideal from a research perspective, imposes unrealistic and additional response burden on both pharmacists and participants and may exclude those consumers who also represent a component of a community pharmacy. Additionally, a definition of LBP was not provided as the intent of the pamphlet was for consumers using the pamphlet in a real world setting to self-identify as having LBP.

Pharmacy staff explained the requirements of the study to participants and obtained written, informed consent from participants at enrolment into the trial. After consenting, all participants were asked to complete a baseline questionnaire (T0) prior to any intervention and prior to leaving the pharmacy, and this was estimated to take 10 minutes to complete. This T0 questionnaire was sealed in a pre-paid envelope and all questionnaires were posted daily to the research team. Each pharmacy was given 15 recruitment packages containing a study information sheet, a consent form, a T0 questionnaire, and the consumer LBP pamphlet (intervention groups only; the control group did not receive the pamphlet).

### Randomisation and Allocation

Pharmacies meeting the inclusion criteria were identified and recruited prior to being randomly allocated to clusters. An index of education and occupation was assigned to each participating pharmacy based on the Australian Bureau of Statistics Socioeconomic Index for Area (SEIFA) [32]. Indices, expressed geographically by Australian postcode, are derived from national census data and are used as a relative ranking between geographic areas, expressed in deciles (1–10). The ascending distribution of indices across the pharmacies (range: 2–10) was divided into thirds, such that a low, medium and high SEIFA block was created. Pharmacies from within each SEIFA block were then randomised (simultaneously) to one of three study cluster groups: two intervention groups (pamphlet+education [n = 11]; pamphlet only [n = 11]; and a control (usual care) group [n = 13]). Therefore, within each group, the SEIFA range was 2–10 representing an equal spread of education and occupation status across the study groups. Randomisation at the pharmacy (cluster) level was chosen as this minimised potential contamination that may have occurred if individual pharmacy consumers were randomised and the pharmacies were required to concurrently manage consumers in both the control and intervention groups. Additionally, the consistency of delivery of the same intervention was considered an important consideration in randomising to pharmacies rather than participants. Allocation of pharmacies was concealed from the PSWA and the investigator (KW) who provided access to the clusters. At the individual level, allocation was unconcealed and participants were informed that they would be involved in a randomised trial, and depending on the pharmacy attended, would be allocated to: (i) receive the pamphlet; or (ii) not receive the pamphlet (control group). The control group were advised that they would receive a copy of the pamphlet upon final completion of the study. To reduce selection bias at the individual level, all participants identified within pharmacy clusters as eligible, were included in the trial.

### Blinding

One investigator (JC) was responsible for generating the random allocation sequence, enrolling clusters and assigning clusters to intervention groups. The other primary investigators (HS, AMB, KW) were not involved in the delivery of the intervention and, as outcomes assessors, were blinded to the cluster allocation until after the independent statistical analysis (AJS) was completed. Due to the nature of the intervention, pharmacies and participants were unblinded to their group allocation, as the pragmatic nature of the study meant that both were aware of the specific intervention being undertaken.

### Intervention

The pamphlet provided evidence-based information about management for LBP (consistent with current recommendations [33]) by highlighting key messages for consumers, such as a need to stay active, and stay positive and stay engaged (for example, at work and socially). Interventions in each trial cluster are summarised below (see also Figure 1 for more detail) and are reported according to recommendations from Consolidated Standards of Reporting Trials (CONSORT) [34] and the CONSORT 2010: extension to cluster randomised trials [31]. The intervention groups were: (i) pamphlet+education: in addition to usual care provided by the pharmacy, participants received verbal reinforcement of the pamphlet’s content from a trained pharmacy staff member; (ii) pamphlet only: in addition to usual care provided by the pharmacy for consumers with LBP, participants were provided with the pamphlet, but without further specific reinforcement of pamphlet content; (iii) usual care only (control: no pamphlet at the time of the trial).

No specific measure of fidelity for pharmacist-delivered messages was used. However, in order to ensure standardisation of the verbal reinforcement that was delivered to participants, pharmacist staff allocated to the pamphlet+education intervention were provided with specific training. This training was conducted as pre-trial workshops (HS), during which pharmacists were instructed about the key pamphlet messages to reinforce and were advised about the necessity of delivering these messages strictly in accordance with the pamphlet content. Pharmacists were encouraged to request clarification and feedback in regards to delivery of the messages at the time of these workshops and throughout the trial if/as required. Pharmacies allocated to intervention groups were encouraged to use their co-operative pharmacy management skills to emphasise the biopsychosocial model of care through reinforcement of the following key messages which related to both acute and chronic LBP: ‘there is a lot you can do yourself to manage your pain’; ‘most people recover fully’; ‘stay active if possible’: ‘moving helps reduce pain’; ‘maintain your usual activities’; ‘stay at work if possible’; ‘stay positive’; ‘avoid prolonged bed rest’; ‘X-rays or other imaging is usually *not* required’. If the consumer’s LBP was acute, pharmacists reinforced the specific messages targeting self-management for acute LBP and if their LBP was chronic, pharmacists reinforced the specific messages targeting self-management of chronic LBP. Pharmacists also indicated to participants, the ‘red flags’ conditions specified in the pamphlet and which required medical review (including severe, constant (24 hours a day) back pain; severe back pain with leg pain and weakness or changes in sensation extending into the leg/s; loss of bowel and bladder control; numbness in the genital area or buttocks; fever; a need for continuous pain-relieving medicine for more than a few days). If consumers identified any of these ‘red flag’ items, or were identified as needing help regarding pamphlet items such as ‘relaxation strategies’ or were feeling ‘anxious, stressed or depressed’, pharmacists were instructed to refer the consumers to their family doctor or health care professional for further advice.

### Outcome Measures

Participants completed questionnaires at 3 time points: baseline (T0), prior to any intervention; two weeks after baseline (T1); and again at 8 weeks after baseline (T2). Questionnaires for T1 and T2 were posted to consumers (residential addresses) with reply-paid envelopes. Non-responders received a single reminder by email or by phone approximately 2–3 days after the T1 or T2 deadline, requesting they complete the survey.

#### Individual-level outcomes

Individual level demographic (age and gender), pain history (pain duration and days off work or higher education) and highest level of education data were collected at T0. Other outcome measures were collected at all three time-points. Beliefs about inevitable consequences of future life with low back problems were measured using the Back Pain Beliefs Questionnaire (BBQ) [35]. The BBQ consists of 14 items each rated on a 5-point Likert scale, scored from 1 (completely disagree) to 5 (completely agree). Scores range between 9 and 45 with lower scores representing more negative beliefs about LBP. The internal consistency (α = 0.70) and test-retest reliability (ICC = 0.87) of the BBQ have been established previously [36]. Fear avoidance beliefs and attitudes related to LBP were measured using the Fear Avoidance Beliefs Questionnaire (FABQ) [37]. The FABQ contains 16 items rated on a 7-point Likert scale, scored as 0–6 (completely agree to completely disagree). Two subscales are calculated: FABQ-physical activity (FABQ-PA) scored out of 24; and FABQ-work (FABQ-W) scored out of 42. Higher scores indicate higher fear avoidance beliefs and attitudes. Internal consistency for FABQ-PA and FABQ-W have been reported as α = 0.77 and α = 0.88, respectively [37]. The BBQ and FABQ scores were selected *a priori* as the primary outcomes. Average LBP severity in the previous 24 hours and activity impairment were measured using an 11 point numerical rating scale (NRS), with anchors at 0 (no pain/no effect on activities of daily living) and 10 (worst pain/unable to perform any activities of daily living) [38], [39]. The perceived usefulness of the pamphlet was scored by participants receiving the interventions (pamphlet with and without education) using a Global Perceived Impression of Usefulness (GPIU) scale which was based on an 11 point NRS anchored at 0 (not at all useful) and 10 (extremely useful) [40], and measured at T1 and T2. Pain severity, activity impairment and usefulness were selected as secondary outcomes.

#### Cluster-level outcomes

At the completion of the study, pharmacists at each pharmacy allocated to an intervention were asked to respond to a number of questions regarding their perceived usefulness of the pamphlet (see result table for questions). The first of these questions was rated using an 11 point numerical rating scale from 0 (not at all useful) to 10 (extremely useful). The next 4 questions were rated using nominal response categories (yes/no/unsure).

### Sample Size Estimate

As explained earlier (see ‘trial design’), initial power calculations were based upon a non-clustered RCT of individuals (Protocol S1) and were based on population responses from an earlier study [41]. Assuming a mean change of 1.9 points on the BBQ, a standard deviation of 5.0 based on a recent survey in WA using the BBQ [10], 80% power and an alpha level of 0.05, a minimum sample size requirement of 110 per group was estimated (N = 330). However, as the study was initiated it became apparent that it was more feasible to randomise by pharmacy rather than by individual, and therefore revised estimates of study power were performed using all baseline data after recruiting 35 pharmacies to participate, to optimise estimation of the intra-pharmacy correlation of back beliefs scores for sample size calculation for the cluster design, as currently recommended [42]. The intra-pharmacy correlation of back beliefs scores was estimated at 0.1, and the between-pharmacy standard deviation at 4. Only data from this study were used to derive ICC estimates, as to our knowledge no other studies with a C-RCT design and employing the same outcome measures were available in the literature. The power of the study to detect minimal clinically important differences in back beliefs (BBQ) of 2 points, with a minimum of 11 pharmacies in each intervention and a sample of 10 consumers from each pharmacy was estimated to be 78%.

### Statistical Analyses

The cluster RCT design required a different analytic approach from our initial RCT protocol (Protocol S1). Demographic and clinical characteristics of the study cohort at cluster and participant levels were summarised using descriptive statistics. Change from baseline was estimated separately in intervention clusters using paired t-tests. The mean effects of intervention on back beliefs and fear avoidance beliefs (physical activity and work-related), pain intensity and activity impairment were estimated using six, three-level linear mixed models with random intercepts for pharmacy and time (2 and 8 weeks), incorporating terms for intervention group, time and intervention group x time interaction, and adjusting for the measure at baseline. This allowed estimation of the effects of intervention separately at both time-points. Two *a priori* contrasts were performed; i) pamphlet with or without pharmacist reinforcement versus usual care and ii) pamphlet with versus without pharmacist reinforcement. Additionally, a similar linear mixed model was used to evaluate differences in satisfaction with the intervention between those groups receiving the pamphlet with or without pharmacist reinforcement. Mean differences and 95% confidence intervals were estimated for these contrasts. Analysis was performed on an ‘available case’ basis, with participants missing data for either one of the 2 or 8 week follow-up included in the models, as the linear mixed model is a likelihood-based estimation procedure resulting in non-biased estimates provided data are missing at random. All outcome measures were examined for normality of distribution, and final models were examined to confirm the absence of unduly influential observations. Statistical analysis was performed using Stata/IC 12.1 for Windows (Statacorp LP, College Station TX USA).

## Results

A total of 35 community pharmacies were recruited for the study from April to May 2011. Three hundred and seventeen consumers provided baseline data from 5 May to 24 August 2011, inclusive. Descriptive statistics for the pharmacies and consumers sampled are provided in Table 1.

**Table 1:** Baseline characteristics of the study cohorts at cluster level (N = 35 pharmacies) and participant level (n = 317).

Characteristic	Groups		
	Pamphlet with education	Pamphlet only	Control (usual care)
n (%) participating consumers	102 (32.2)	111 (35.0)	104 (32.8)
N (%) participating pharmacies	11 (31.4)	11 (31.4)	13 (37.2)
Mean (min, max) n per pharmacy	9.3 (1,22)	10.1 (2,25)	8.0 (2,33)
SEIFA of pharmacies	3–10	2–10	2–10
N (%) female	57 (55.9)	72 (64.9)	63 (60.6)
Mean (SD) age; range [years]	43.3 (13.2); 18–65	44.2 (12.7); 19–65	44.3 (11.8); 20–64
Duration of current LBP episode. N (% within group)			
<3 months	20 (19.6)	15 (13.5)	24 (23.1)
≥3 months intermittently	32 (31.4)	34 (30.6)	23 (22.1)
≥3 months continuously	50 (49.0)	61 (55.0)	57 (54.8)
*No response*	0	1 (0.9)	0
Time loss off work/education for current episode of LBP			
0 days	42 (41.2)	56 (50.5)	50 (48.1)
1–2 days	17 (16.7)	10 (9.0)	14 (13.5)
3–7 days	10 (9.8)	11 (9.9)	8 (7.7)
8–14 days	7 (6.9)	3 (2.7)	4 (3.8)
15–30 days	4 (3.9)	1 (0.9)	2 (1.9)
1–2 months	2 (2.0)	3 (2.7)	3 (2.9)
2–3 months	2 (2.0)	2 (1.8)	0 (0)
3–6 months	4 (3.9)	1 (0.9)	3 (2.9)
6–12 months	2 (2.0)	3 (2.7)	1 (1.0)
>1 year	9 (8.8)	16 (14.4)	15 (14.4)
*Missing*	3 (2.9)	5 (4.5)	4 (3.8)
Highest level of education			
Some secondary school	11 (10.8)	11 (9.9)	8 (7.7)
Completed secondary school	27 (26.5)	26 (23.4)	21 (20.2)
Trade certificate(s) or diploma(s)	32 (31.4)	36 (32.4)	38 (36.5)
University degree(s)	31 (30.4)	37 (33.3)	35 (33.7)
*Missing*	1 (1.0)	1 (0.9)	2 (1.9)
24 hour pain severity^a^ (range: 0–10). Mean (SD); range	5.2 (2.4); 0–10	5.0 (2.3); 0–10	5.7 (2.0); 2–10
24 hour activity impairment^b^ (range: 0–10). Mean (SD); range	4.2 (2.3); 0–10	4.3 (2.7); 0–10	4.9 (2.7); 0–10
Back beliefs^c^ (range 9–45). Mean (SD); range	25.8 (7.3); 9–45	25.7 (7.5); 9–42	25.0 (6.6); 12–38
Physical activity–related fear beliefs^d^ (range 0–24). Mean (SD); range	15.1 (5.3); 1–24	15.7 (5.3); 2–24	15.7 (6.1); 0–24
Work-related fear beliefs^d^ (range 0–42). Mean (SD); range	17.2 (12.0); 0–42	17.9 (11.9); 0–42	17.5 (12.5); 0–42

a measured with numerical rating scale, with possible score ranging from 0 (“no pain”) to 10 (“worst pain”).

b measured with numerical rating scale, with possible score ranging from 0 (“no effect on activities of daily living”) to 10 (“unable to perform any activities of daily living”).

c measured using the Back Beliefs Questionnaire (BBQ), with possible score ranging from 9 to 45 with higher scores indicating more positive beliefs.

d measured using Fear Avoidance Beliefs Questionnaire, with possible score ranging from 0 to 24 for physical activity-related fear and 0 to 42 for work-related fear. Higher scores indicate higher fear avoidance beliefs and attitudes.

Of the 35 pharmacies recruited (317 consumers), no clusters were lost to follow up (Figure 1). At an individual level, 107 participants were excluded from the analysis as no follow up data were available at either 2 or 8 weeks. On an available case basis, 210 cases were available for analysis in the linear mixed models (n = 24 were followed up at 2 weeks only; n = 38 were followed up at 8 weeks only; n = 148 were followed up both time points, resulting in n = 148+24+38 = 210 analysed).

The proportion of non-responders (n = 107) was similar across the intervention groups (pamphlet plus education 32.9%, pamphlet only 39.3%, control 29.9%). There were no significant differences between responders and non-responders for any of the baseline measures of outcome variables, or for sex, pain duration, activity impairment or education level. However, non-responders (mean age 39.8 years) were significantly younger than responders (mean age 46.5 years, mean difference −7.7 yrs, 95%CI: −10.5 to −4.9 yrs).

### Back Beliefs

**Figure 2:**
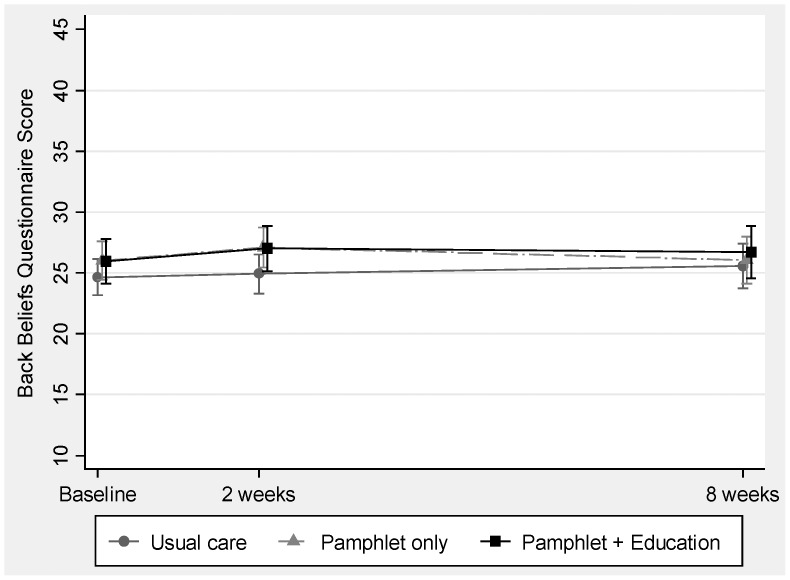
Back belief scores (BBQ) are shown for responders in intervention and control (usual care) groups. Values shown are unadjusted means (i.e.; including baseline estimates) with 95% confidence intervals. Measures were obtained at baseline, 2 and 8 weeks, but data are slightly offset for clarity. Higher scores represent more positive beliefs.

There were only small and statistically non-significant improvements in back beliefs estimated in all three groups at 2 weeks (control; 0.1 (95%CI: −0.9 to 1.1), pamphlet only 1.2 (95%CI: 0 to 2.4), pamphlet plus education 0.7 (95%CI: −0.5 to 2.0)), and at 8 weeks (control; 1.0 (95%CI: −0.4 to 1.3), pamphlet only 0.5 (95%CI: −0.9 to 2.0), pamphlet with education 0.8 (95%CI: −0.6 to 2.3)). After adjusting for baseline scores, there were no significant differences in back beliefs at 2 or at 8 weeks between pamphlet (with or without education) versus control (usual care), or between pamphlet with versus without education (Figure 2 and Table 2). The within-pharmacy correlation of back beliefs was estimated to be 0.046 (95%CI: 0.007 to 0.252).

**Table 2:** Estimated effects of pamphlet (with or without education) versus usual care (control) and effects of pamphlet with education versus pamphlet without.

	Pamphlet withEducation (PE)	Pamphlet Only (PO)	Control (C)	Adjusted intervention effect (PE&PO - C)	*P value*	Adjusted interventioneffect (PE - PO)	*P value*
Back beliefs^a^ (n = 206)							
2 weeks	27.0 (7.4)	27.1 (6.3)	24.9 (6.6)	1.3 (−0.3 to 2.9)	0.109	−0.6 (−2.4 to 1.2)	0.520
8 weeks	26.7 (8.1)	26.1 (7.0)	25.8 (6.8)	0.4 (−1.2 to 2.0)	0.640	0.4 (−1.5 to 2.3)	0.668
Physical activity-related fear^b^ (n = 206)							
2 weeks	15.1 (5.8)	13.7 (5.5)	15.0 (5.5)	0.2 (−1.2 to 1.6)	0.762	1.3 (−0.4 to 2.9)	0.143
8 weeks	13.8 (6.4)	13.4 (5.8)	14.8 (4.9)	−0.6 (−2.0 to 0.9)	0.462	0.5 (−1.2 to 2.2)	0.591
Work-related fear^b^ (n = 203)							
2 weeks	15.9 (12.4)	17.6 (11.07)	18.6 (12.2)	−1.5 (−3.5 to 0.6)	0.161	−0.7 (−3.2 to 1.7)	0.566
8 weeks	15.4 (10.9)	15.6 (11.3)	17.7 (12.8)	**−2.3 (−4.4 to −0.2)**	**0.034**	−0.2 (−2.7 to 2.3)	0.864
Pain severity^c^ (n = 210)							
2 weeks	4.3 (2.3)	4.7 (2.1)	4.3 (2.4)	0.5 (−0.1 to 1.2)	0.107	−0.3 (−1.1 to 0.5)	0.448
8 weeks	3.7 (2.6)	4.3 (2.5)	4.4 (2.5)	−0.2 (−1.1 to 0.5)	0.613	−0.7 (−1.6 to 0.1)	0.089
Activity impairment^d^ (n = 210)							
2 weeks	3.4 (2.5)	3.7 (2.1)	3.6 (2.8)	0.3 (−0.3 to 1.0)	0.312	0.0 (−0.8 to 0.8)	0.935
8 weeks	3.1 (2.7)	3.5 (2.5)	3.7 (2.7)	−0.2 (−0.9 to 0.5)	0.520	−0.2 (−1.0 to 0.6)	0.637

a measured using the Back Beliefs Questionnaire (BBQ), with possible score ranging from 9 to 45 with higher scores indicating more positive beliefs.

b measured using Fear Avoidance Beliefs Questionnaire, with possible score ranging from 0 to 24 for physical activity-related fear and 0 to 42 for work-related fear. Higher scores indicate higher fear avoidance beliefs and attitudes.

c measured with numerical rating scale, with possible score ranging from 0 (“no pain”) to 10 (“worst pain”).

d measured with numerical rating scale, with possible score ranging from 0 (“no effect on activities of daily living”) to 10 (“unable to perform any activities of daily living”) Data represent adjusted means.

### Beliefs Regarding the Effect of Physical Activity on LBP (FABQ-pa)

**Figure 3:**
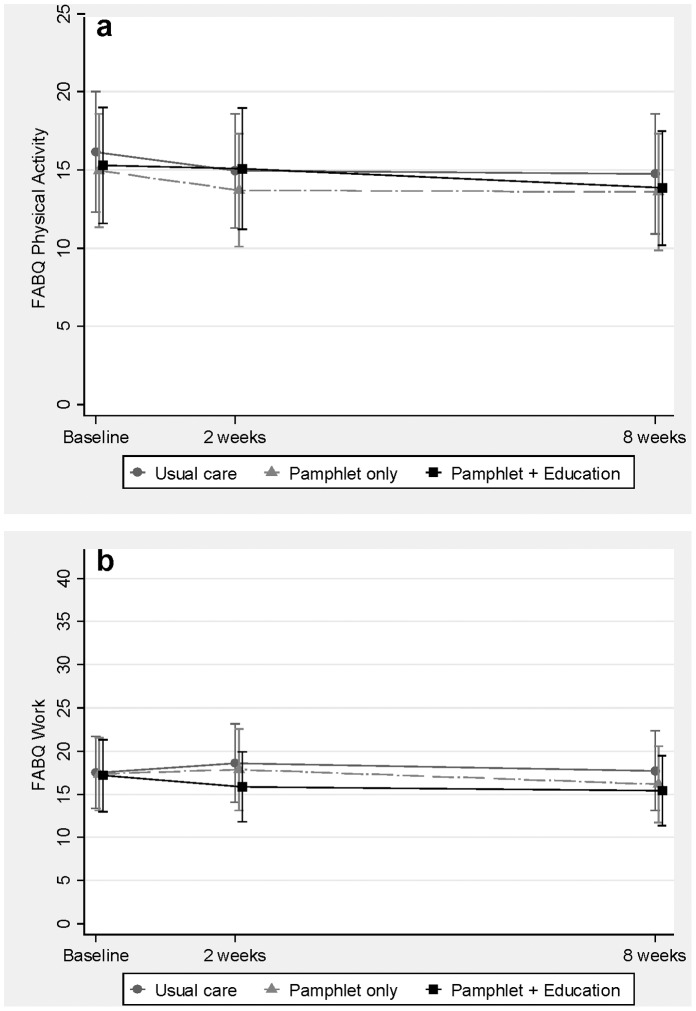
Beliefs related to (a) physical activity (FABQ-pa) and (b) work (FABQ-w) are shown. Graphs represent responder data for the intervention and control (usual care) groups. Values shown are unadjusted means (i.e.; including baseline estimates) with 95% confidence intervals. Measures were obtained at baseline, 2 and 8 weeks, but data are slightly offset for clarity. Higher scores indicate higher fear avoidance beliefs and attitudes.

There was a statistically significant decrease in physical activity-related fear at 2 weeks in the control (usual care) group (−1.3, 95%CI: −2.4 to −0.2), but not in the pamphlet only group (−1.3, 95%CI: −2.8 to 0.3) or the pamphlet with education group (0.0, 95%CI: −1.4 to 1.4). At 8 weeks there was a significant decrease in physical activity-related fear from baseline in the control group (−1.8, 95%CI: −3.0 to −0.6) and the pamphlet only group −2.0 (95%CI: −3.5 to −0.7), but not the pamphlet with education group (−1.5, 95%CI: −3.0 to 0.1). After adjusting for baseline scores, there were no significant differences in physical activity-related fear at 2 or 8 weeks between pamphlet (with or without education) versus control, or between pamphlet with versus without education (Fig 3 (a) and Table 2). The within-pharmacy correlation of physical activity-related fear was estimated to be negligible (<0.001).

### Beliefs Regarding the Effect of Work on LBP (FABQ-w)

There were no significant changes in work-related fear in all three groups at 2 weeks (control; 0.9 (95%CI: −0.6 to 2.4), pamphlet only 0.0 (95%CI: −2.0 to 2.1), pamphlet with education −0.7 (95%CI: −2.3 to 0.8). There was no change from baseline in work-related fear in the control group at 8 weeks (0.6, 95%CI: −1.0 to 2.3). Although there was a decrease from baseline in work-related fear in the pamphlet only and pamphlet with education groups at 8 weeks, these improvements were not statistically significant (pamphlet only −1.4 (95%CI: −3.6 to 0.9); pamphlet with education −1.5 (95%CI: −3.4 to 0.3). However, after adjusting for baseline scores, work-related fear was significantly lower in those consumers receiving pamphlet (with or without education) versus control (difference −2.3, 95%CI: −4.4 to −0.2, Table 2), but there was no significant difference between pamphlet with versus without education (Fig 3 (b) and Table 2). The effect size of this change was small (0.2) and, to our knowledge, there is no established minimal clinically important difference for this outcome. The within-pharmacy correlation of work-related fear was estimated to be 0.016 (95%CI: 0.001 to 0.378).

### Pain Severity

There was a statistically significant decrease in pain severity at 2 weeks in the control group (−1.3, 95%CI: −1.9 to −0.8), but not in the pamphlet only group (−0.5, 95%CI: −1.0 to 0.1) or the pamphlet with education group (−0.6, 95%CI: −1.2 to 0.1). At 8 weeks there was a significant decrease in pain severity from baseline in the control group (−1.0, 95%CI: −1.7 to −0.4) and the pamphlet with education group −1.5 (95%CI: −2.2 to −0.7), but not the pamphlet only group (−0.7, 95%CI: −1.4 to 0.0). After adjusting for baseline scores, there were no significant differences in pain severity at 2 or 8 weeks between pamphlet (with or without education) versus control, or between pamphlet with versus without education (Table 2). The within-pharmacy correlation of pain severity was estimated to be negligible (<0.001).

### Activity Impairment

There was a statistically significant decrease in activity impairment at 2 weeks in the control group (−1.3, 95%CI: −1.8 to −0.7), but not in the pamphlet only group (−0.7, 95%CI: −1.4 to 0.0) or the pamphlet with education group (−0.5, 95%CI: −1.1 to 0.1). There were statistically significant reductions in activity impairment in all three groups at 8 weeks (control; −0.8 (95%CI: −1.6 to −0.1), pamphlet only −0.8 (95%CI: −1.5 to −0.1), pamphlet with education −0.9 (95%CI: −1.7 to −0.2). After adjusting for baseline scores, there were no significant differences in disability at 2 or 8 weeks between pamphlet (with or without education) versus control, or between pamphlet with versus without education (Table 2). The within-pharmacy correlation of disability was estimated to be negligible (<0.001).

### Perceived Usefulness of the Pamphlet

Pharmacy consumers who received the pamphlet with education reported a mean GPIU of 6.2 (SD 2.5) at T1 and 5.7 (SD 2.7) at T2, whereas those who received the pamphlet only reported lower mean GPIU of 5.3(SD 2.1) at T1 and 4.9 (SD 2.5) at T2, and although the estimated difference between the two groups was not significant at either time-point (0.9; 95%CI:−0.1 to 1.9 at both T1 and T2), there was weak evidence for a difference pooled over the two time points (0.9∶95%CI:0 to 1.8). At the cluster level, for those pharmacies delivering the pamphlet intervention, pharmacists’ responses to their perceptions regarding the usefulness of the pamphlet for consumers with LBP and for their usual pharmacy practice and future use, are summarised in Table 3. The response rate for this questionnaire for pamphlet with education was 91% (n = 10/11) and for pamphlet only was 82% (n = 9/11).

**Table 3:** Pharmacists’ perceptions of the usefulness of the pamphlet for the intervention clusters (N = 19).

Question	Pamphlet with education (n = 10/11)	Pamphlet only(n = 9/11)
Please rate your perceived usefulness of the pamphlet for consumers with LBP [(0–10; mean (SD)]	7.1 (1.8)	7.4 (0.9)
Do you think the consumers found the pamphlet useful? N(%) Yes/No/Unsure		7 (77%)
		0 (0%)
		2 (22%)
Do you think the consumers found the pamphlet plus education useful? N(%) Yes/No/Unsure	7 (70%)	
	2 (20%)	
	1 (10%)	
Do you think that the ‘pamphlet with education’ was more useful than your usual care?N(%) Yes/No/Unsure	8 (80%)	
	1 (10%)	
	1 (10%)	
Would you use this low back pain education pamphlet in conjunction with yourusual pharmacy low back pain care program in the future? N(%) Yes/No/Unsure	7 (70%)	8 (89%)
	1 (10%)	0 (0%)
	1 (10%)	1 (11%)

## Discussion

Using a cluster-randomised trial, we determined that a pharmacy level intervention implementing a consumer-oriented LBP pamphlet compared to usual community pharmacy care was effective at an individual level, in improving work-related fear beliefs at eight week follow up in those participants receiving the pamphlet intervention (with or without education). The pamphlet delivered with additional education was not more effective in improving beliefs than the pamphlet alone, although consumers’ satisfaction was greater where the pamphlet was provided with additional education from the pharmacist. General LBP-related beliefs, physical activity-related fear beliefs and secondary outcomes related to pain and activity impairment failed to demonstrate any significant improvement. The pamphlet intervention was feasible to implement in community pharmacies in a metropolitan primary care setting and pharmacists in the active intervention groups supported the use of the pamphlet in conjunction with their usual pharmacy care.

### Main Findings

Beliefs in relation to back pain are complex and therefore providing clear, simple evidence-based messages to help improve beliefs for consumers with LBP is considered important, as unhelpful beliefs can significantly contribute to activity impairment and ongoing disability [43]. Moreover, unhelpful beliefs are associated with the development of chronicity [35], [44]. Our findings indicated that the delivery of simple evidence-based messages in the form of an educational pamphlet generated a significant improvement in fear-related beliefs regarding the effect of work on LBP, regardless of whether these messages were verbally reinforced or not. The pamphlet information about work was quite specific about trying to ‘stay at work’, even if modifications were required and it is possible that this specificity was a significant factor in explaining this primary outcome. The effect size of this improvement was modest (0.2) and, to our knowledge, given there is no established minimal clinically important difference for this outcome, it is difficult to predict if or how this primary outcome might impact any longer term work-related disability or potentially influence health economic outcomes [45], such as service utilisation, absenteeism, or presenteeism. However, given that LBP primarily affects the working population and work absenteeism creates a massive threat to human capital [4], [46] the use of a relatively inexpensive evidence-based pamphlet to help improve work-related fear avoidance beliefs, would appear to be a simple and positive component of a health intervention for consumers with LBP. The benefits of a mass media campaign that provided similar evidence-based messages at a population level, is evidenced by the significant improvements in both community and physician beliefs and the associated decline in number of workers’ compensation back claims and health utilization over the duration of, and sustained at 3 years beyond, the campaign [23]. Although not the intent of the current study, extending our research to explore if such a modest change in work-related beliefs would also translate into similar workers’ compensation and health utilization outcomes is recommended and feasible in Australia using linked data. However, any such interpretation must be tempered by an acknowledgement of the lack of improvement in general beliefs about back pain or physical activity-related beliefs and in pain or disability in this trial. In this regard, it is important to highlight that consumers may require more targeted and specific messages and additional components of care in order to improve their general and physical activity-related beliefs and to improve their function and reduce disability [47], [48]. Such an approach may be more important in a cohort of people experiencing persistent LBP, (a significant percentage of our cohort), where potentially more complex factors may be associated with persistent pain. This argument is consistent with the findings from a recent systematic review [49], indicating that educational interventions by pharmacists were not effective in moderating pain intensity and interference with daily life for consumers with persistent LBP, but were effective for consumers with subacute LBP provided the educational interventions were intense (2.5 hours). Previously, we have also shown that it is possible to generate transient improvements in back pain beliefs among consumers with persistent LBP using a short 6 hour evidence-based face-to-face intervention, however this improvement was not sustained at 3 months follow up [48]. In order to both facilitate improved consumer beliefs and for this improvement to be associated with more positive health behaviours (for example, reduced activity impairment/disability), most likely requires an individualised, multimodal approach to management, incorporating specific skills (i.e. ‘doing’: for example, the skill of pacing activity) rather than solely providing simple evidence-based knowledge (‘knowing’: avoiding prolonged bed rest) especially for more complex, persistent LBP [50]. It is possible that if the pamphlet messages were delivered in a more comprehensive framework incorporating a skills component, as previously outlined [50], the work-related beliefs outcomes may be even better. A similar approach employing both knowledge and skills, was successfully implemented for a community based and pharmacist-led multidisciplinary intervention to identify knee osteoarthritis cases, with significant outcomes including improved function, pain, and quality of life [19]. However, the effectiveness of such an approach would also depend on the capacity of the health workforce to impart those skills in a consistent and evidence-informed manner, and depend also on the health literacy of consumers (i.e.; their capacity to understand the knowledge and their capacity to use those skills) [51].

The ability to initiate changes in LBP-related beliefs and behaviours may also vary according to the clinical setting and target population, the geographic location, the typical clinical practice behaviours of health professional, and the type(s) of consumer behaviours associated with LBP [52]. In our study, the cohort sampled was purposively heterogenous to reflect a typical Australian metropolitan, community pharmacy population with LBP. However, it is possible that a sub-grouping approach, for example as proposed by Hill et al [53], or a temporal classification (acute or chronic) of LBP symptoms, where patients are better matched via classification to timely, appropriate interventions [50] may provide different outcomes, although such an approach is probably impractical in a community pharmacy setting. The effectiveness of any campaign may also depend on the specific messages and who delivers those messages [52]. In Australia, a mass media campaign targeting the general community and health professionals with positive messages about back pain employed different messengers to target different groups such as health professionals, employers, and employees. This campaign resulted in significant sustained improvements in population beliefs about back pain (BBQ and FABQ) that were still observed 3 years later [54]. Using a similar approach of matching of messengers to messages, adapting the key pamphlet messages to better fit the context of a community pharmacy may be more appropriate: for example, by focussing on the appropriate use of medications to create a therapeutic window (i.e.; one created by the use of appropriate analgesia) during which sensibly paced activity (physical activity) could be increased and staying at work encouraged. As patients want written information tailored to them and do not want this to be a substitute for spoken information [55], such as approach to LBP self management may be well suited to a community pharmacy setting. In this setting, optimizing the use of a therapeutic window in order to encourage the adoption of positive health behaviours such as increasing active self management, may be important as beliefs and behaviour for consumers with persistent LBP do not necessarily match [10]. A further consideration may relate to the ability of consumers to effectively seek out information (in this case, through the pharmacy) and understand the information (the simple evidence-based messages in the pamphlet), but lack the skills required (the actual ‘doing’ part) to effectively implement this evidence-based information – the three elements of health literacy [56]. In this regard, we have previously reported that people with LBP have more trouble in engaging in positive lifestyle behaviours compared to those without LBP [51].

Our study outcomes may also have been influenced by patient expectations [57]. Patients can report high levels of satisfaction despite inadequate pain management [58], and expectations may change with accumulating experience [59] as the expectation of a cure is reassessed and downgraded in likelihood, especially by the person living with persistent or recurrent LBP (a significant percentage of our cohort). Therefore, while consumers receiving the pamphlet with education reported higher perceived usefulness (i.e.; reasonably useful) than those that received pamphlet only, and these findings are consistent with previous pharmacy educational interventions for chronic pain [49], there may be little association between this outcome and improved beliefs especially if people already have positive beliefs. The relationship between patients’ expectations and beliefs, the pattern of their pain, and the patient–provider relationships are complex and can determine the level of patient satisfaction for interventions [57], therefore highlighting the need for a such an educational intervention to be considered as providing a component of care, rather than being considered as a sole intervention.

In the context of LBP management, the community pharmacy provided an obvious portal to pilot the dissemination of the LBP educational pamphlet intervention. Community pharmacy has potential as a health promotion setting, due to accessibility, high volume, the respect afforded to pharmacists [60] and the specific need for enhanced health related services above those normally available in community pharmacy for medicines [61]. Additionally, the pamphlet intervention was specifically designed for use in primary care with messages that could be easily reinforced to consumers, consistent with evidence suggesting that outcomes for patients with LBP may be improved when written health information is reinforced by a health professional [24], [62]. Here the importance of upskilling pharmacists to deliver the pamphlet intervention was pre-empted by implementing a short pre-trial workshop explaining the way in which pharmacists could reinforce the key LBP pamphlet messages. Given the findings that pharmacists’ awareness of written information that supports evidence based self-management of LBP is low [63] and recent data which indicate that graduating pharmacists could improve their LBP-related beliefs and clinical recommendations [64], this upskilling was considered important. However, pharmacists may require more comprehensive training to enhance their ability and confidence to support consumer co-management of LBP. So, while community pharmacy can feasibly provide an important role as a primary health care conduit, given the complex and multidimensional nature of persistent LBP, we suggest that a co-management role be viewed as part of a broader community of practice approach to LBP management [12]. An expanded community of practice approach in primary care would broaden the reach of interprofessional care as recommended in current health policy related to pain management [5], [65], and consistent with recommendations for primary care management of LBP [33], with the potential for improved patient outcomes. Such an approach appears feasible given that a majority of pharmacists perceived the pamphlet to be moderately to very useful and more so than usual care, indicated that they would use the pamphlet in future and perceived that consumers with LBP also found the pamphlet useful.

### Strengths and Llimitations

The strengths of this study are reflected in the translation and rigorous evaluation of state health policy into practice, with the involvement of community pharmacy in the dissemination of evidence-based, consistent, cross-discipline messages about LBP [5], [21], [22]. This pragmatic, partnership-based approach was considered a strategic method to overcoming some of the known system barriers to implementation and uptake of clinical guidelines in primary care [1], [30], [50]. Since such clinically-oriented research is typically complicated by time and cost constraints, by what is sustainable and optimal for both high quality research and for real-world clinical research, the shift to a pragmatic cluster trial design enabled better engagement of community pharmacy practices and recruitment of community pharmacy consumers compared to a randomised controlled trial. A further strength relates to the trial design which involved a large number of clusters per group [66] (n = 11–13 per arm; 35 in total). The large sample size was reflective of the general community with LBP and this cohort demonstrated clinical characteristics comparable with other studies from primary care settings [10], [41], [67]. Nonetheless, limitations in the interpretation and generalizability of our findings to other primary care populations of consumers with LBP must be acknowledged. Selection bias cannot be excluded as the pharmacy clusters recruited may have been particularly committed to being involved in delivering the intervention and individual participants self-referred to the study and their motivations for participating may differentiate them from other consumers with LBP not so inclined to participate. As pharmacy proprietors were recruited through a professional association (PSWA), a population bias is possible as some pharmacists may not be members, although current membership data indicate a level of approximately 80%. Given that non-responding participants were significantly younger than responders, we cannot generalise the outcomes to a younger age group. While a limitation, the use of open allocation enabled clinical equipoise to be achieved with the advantages of efficient recruitment and consistent delivery of a single intervention by pharmacists. This streamlined the administration of the interventions (pamphlet with or without education) and ensured sufficient pharmacies were recruited and that the subsequent recruitment of individuals was also more efficient. Additional factors that require consideration include: consumer and pharmacist data were based on self-report measures captured at limited time points; evaluation of compliance and consistency by pharmacists in reinforcing the pamphlet messages was not practical given the busy nature of a community pharmacy. Whilst a substantial proportion (107 of 317 (33.8%)) of consumers did not respond to either the 2 or 8 week mail out after a reminder email or phone call, this proportion was similar in the three intervention clusters, limiting the potential for bias of effect estimated due to responder bias. Data from participants within the same pharmacy are potentially non-independent, however estimates of the within-pharmacy correlations of the primary outcome measures from this study were very low, so the sample size was adequate to detect differences of approximately half a standard deviation in outcome measures, but not adequately powered to detect smaller changes. Direct consumer incentives may also be considered as a useful strategy to boost consumer response rates in pharmacy practice research with the caveat that ethical requirements are not compromised. Participants within the same cluster were not truly independent, so the effective sample size is less than the actual number of participants, hence the original sample size estimate would be inadequate for a cluster design. Patient expectations which may influence perceived benefits and beliefs about persistent pain [57] were not measured but may provide further informative data. The potential effectiveness of the pamphlet on general and physical activity fear-related beliefs may be better explored using additional qualitative approaches or measuring other relevant domains (such as self efficacy and locus of control), which may better align with the pamphlet messages.

### Conclusion

The use of community pharmacies as a primary care portal for the implementation of evidence-based information to consumers with LBP is feasible and the pamphlet intervention was effective in improving consumers’ work-related fears about LBP at eight weeks. Consumers with LBP and pharmacists perceived the pamphlet to be useful, and pharmacists reported that they would use the pamphlet in conjunction with their usual pharmacy care program. Further research is required to examine whether this implementation would be more effective in improving more general beliefs and physical activity related beliefs, quality of life and costs if supported by an expanded interdisciplinary community of practice to better address the complex multidimensional nature of LBP.

## Supporting Information

Checklist S1 **CONSORT Checklist.**
(DOCX)

Protocol S1 **Trial Protocol.**
(PDF)
