# Supplementary material for: Translating Evidence for Low Back Pain Management into a Consumer-Focussed Resource for Use in Community Pharmacies: A Cluster-Randomised Controlled Trial
Source: PLoS One. 2013 Aug 20;8(8):e71918. doi: 10.1371/journal.pone.0071918 (PMC3748095; doi:10.1371/journal.pone.0071918)
Supplement: Protocol S1 — Trial Protocol. (PDF) [file pone.0071918.s002.pdf]

PROJECT PROPOSAL

Background

The efficacy of social marketing campaigns in facilitating a societal shift in beliefs and attitudes towards low back pain (LBP) is well established. Evidence suggests that campaigns need to be exhaustive and utilise multimedia. The Spinal Pain Model of Care recommends the use of social marketing strategies in WA, specifically mass media campaigns. Development of a single, Government-issued resource which delivers simple, evidence-based messages to the public about active pain management strategies for LBP is the first step in developing a suite of social marketing resources for minimising the burden of spinal pain in WA.

The Spinal Pain Implementation Working Group (SPIWG) has developed a consumer pamphlet which contains evidence-based information about the course of, and self management strategies for, acute and chronic LBP. The pamphlet will be aimed at individuals who seek care for, or information about, LBP in primary care settings (eg pharmacies, GP practices, allied health practices).

The pamphlet has undergone a period of consultation with key professional health bodies and health stakeholders. The pamphlet content is supported by the following professional bodies: Australian Clinical Psychology Association; Australian and New Zealand College of Anaesthetists; Australian Osteopathic Association; Australian Pain Society; Australian Physiotherapy Association; Australian Rheumatology Association (WA); Chiropractors' Association of Australia; Health Consumers' Council; Pharmaceutical Society of Australia (WA); and the Royal Australian College of General Practitioners. Providing the opportunity for the professional bodies of different disciplines to endorse the pamphlet content encourages inter-professional consistency in the delivery of evidence-based information to individuals who seek information about spinal pain. Additionally, messages are targeted towards best-practice *self-management* of spinal pain, where appropriate. Where additional support is required, recommendations to seek evidence-based guidance by health professionals are encouraged.

Aim

The aim of this project is to undertake an evaluation of the LBP pamphlet within a primary care context. The SPIWG will partner with Curtin University and the Pharmaceutical Society of Australia (WA) (PSA-WA) for the evaluation. The project will determine whether providing the pamphlet to individuals who visit a community pharmacy to purchase pain-relieving medications for LBP is effective in improving beliefs about LBP and minimising fear avoidance behaviours related to LBP, compared to not providing the pamphlet. A secondary aim is to determine whether providing the pamphlet with the pharmacist reiterating the key messages to the consumer is more effective than providing the pamphlet alone.

Design

A randomized, controlled trial, consisting of 3 study arms (A, B and C), will be undertaken in partnership with 20 community pharmacies in the Perth metropolitan area. A convenience sample of pharmacies will be selected through the PSA-WA. Participating pharmacies will cover major zones of the Perth metropolitan area to represent a range of socioeconomic levels. Ten pharmacies will be involved in both the 'control' (A) and 'pamphlet only' (B) arms while a separate ten pharmacies will be involved in the 'pamphlet with education' (C) arm only. Pharmacies will be allocated to study groups randomly. Each pharmacist who participates in the workshop, will receive training from the evaluation team according to their allocated study arm and remuneration for recruitment.

Participants

Pharmacists and/or pharmacy staff will identify individuals who present to the pharmacy to purchase pain-relieving medications for LBP by:

i) asking customers about the reason for their medication purchase or GP medicine prescription;

ii) answering questions from customers about medication options for LBP or;

iii) customers approaching pharmacy staff about the study in response to posters placed in the pharmacy which advertise the study.

Any pharmacy customer aged 18-70 years who is currently experiencing low back pain and has adequate understanding of written and spoken English is eligible to participate. There are no exclusion criteria for this study. Each pharmacy will recruit 12 consecutive customers who meet the inclusion criteria for each study arm. The total sample size will therefore be N=360. Customers who are interested in participating will be asked to complete a consent form and a baseline questionnaire. The completed form and questionnaire will be sent by the pharmacy to Curtin University in a reply paid envelope.

Outcome measures

Data will be collected at baseline (T0) and then at 2 weeks (T1), and 8 weeks (T2) after the intervention. Table 1 shows which outcomes measures will be measured at particular time points. Beliefs about back pain will be collected using the Back Pain Beliefs Questionnaire and fear avoidance behaviours will be collected using the Fear Avoidance Beliefs Questionnaire. Pain intensity and disability will be measured using an 11 point numeric rating scale (NRS). Demographic (age, gender, highest level of education) and pain history characteristics (duration of symptoms) will be measured at baseline only while an 11 point NRS for Global Perceived Impression of Usefulness will be used at time points subsequent to baseline to measure the usefulness of the pamphlet.

Table 1: Outcome measures assessed by time point.

**Instrument**	**T0**	**T1**	**T2**
Demographics	✓		
Pain history	✓		
BPBQ	✓	✓	✓
FABQ	✓	✓	✓
Pain intensity	✓	✓	✓
GPIU		✓	✓
Disability	✓	✓	✓

Participants will be asked to provide informed consent at baseline, including their contact details and date of birth. All data will be collected using a 2 page, teleform paper questionnaire which will be de-identified but linked to person details using a unique study identifier. Data at T0 will be collected onsite at the pharmacy while follow-up questionnaires (T1, T2) will be posted to participants with a reply-paid envelope. All data will be returned to Curtin University for secure storage and analysis. Participants who do not return follow-up questionnaires will be contacted by a project officer and reminded to do so.

Interventions

Interventions will be phased in the following sequence:
A) Control (pharmacies 1-10): pharmacists will be asked to invite the first 12 customers who meet the inclusion criteria to participate in the study as a control (N=120). In the control arm, customers will not receive any information about self-management for LBP (i.e.; will receive 'usual care' only).

B) Pamphlet only (pharmacies 1-10): pharmacists will be asked to invite the next 12 customers, that is the next 12 customers after the control participants have been recruited, who meet the inclusion criteria, to participate in the study (N=120). In this arm, participants will receive a pamphlet from the pharmacist without any further information about self management, other than usual instructions from the pharmacist relating to medication intake, dosage and side-effects. Baseline data will be collected *prior to* the pamphlet being provided.

C) Pamphlet with education (pharmacies 11-20): pharmacists will be asked to invite the first 12 customers who meet the inclusion criteria to participate in the study (N=120). In this arm, participants will receive a pamphlet from the pharmacist *and* the pharmacist will review the content of the pamphlet with the participant. Information provided by pharmacists will be standardised. Baseline data will be collected *prior to* the pamphlet and further information being provided. The role of the pharmacist will be to reinforce the key messages contained in the pamphlet

Analysis

A 3x3 ANOVA with one repeated measure will be used to examine main effects for group (k=3) and time point (k=3), as well as interactions between the factors.

Ethical implications

Approval to conduct the study will be sought from the Human Research Ethics Committee of Curtin University and the Department of Health (WA).

Staffing

One project officer will be appointed at level 4.1 (Curtin General Staff salary level) at 0.2 FTE for 6 months to coordinate the study and manage the data collection, data entry and data storage processes. Data analysis and report writing will be undertaken by Honours students in Pharmacy and/or Physiotherapy degrees at Curtin University. Overall management of the study will be undertaken by a member of the project team (yet to be confirmed).

Feasibility

Using a partnership arrangement between Health Networks, Curtin University, Musculoskeletal Health Network and the Pharmaceutical Society of Australia, completion of data collection is feasible within 6 months with a budget of $14,300. It is envisaged that recruitment of participants from 20 pharmacies for each arm of the study will be possible within 4 week blocks; ie 12 participants per pharmacy per 4 week block (3 customers per week). Providing a modest remuneration to each pharmacy will also assist with recruitment.

Budget

Photocopying and postage ($5 per person)	$1,500
Design and printing of advertising posters	$1,000
Design and printing of teleform sheets for data collection	$1,000
Design and printing of 500 pamphlets	$500
Training of pharmacists (evening forum)	$500
Project officer (0.2 FTE 6 months, level 4.1 with oncost)	$6,200
Remuneration to pharmacies ($10 per recruit)	$3,600

Total **$14,300**

Appendix 1: Sample size estimates

BBQ: assuming a mean change of 1.9 points (based on population response in Victoria from Buchbinder et al 2001), STDEV 5.0 (based on WA survey) and 80% power and alpha 0.05, sample of 110 per group needed (N=330).

FABQ-phys: assuming a mean change of 1.5 points (based on population response in Victoria from Buchbinder et al 2001), STDEV 5.0 (based on WA survey) and 80% power and alpha 0.05, sample of 176 per group needed (N=528).

REFERENCES

1. Buchbinder R. Self-management education en masse: effectiveness of the Back Pain: Don't Take It Lying Down mass media campaign. Med J Aust 2008;189:S29-32.

2. Buchbinder R, Jolley D, Wyatt M. 2001 Volvo Award Winner in Clinical Studies: Effects of a media campaign on back pain beliefs and its potential influence on management of low back pain in general practice. Spine (Phila Pa 1976) 2001;26:2535-42.

3. Buchbinder R, Gross DP, Werner EL, Hayden JA. Understanding the characteristics of effective mass media campaigns for back pain and methodological challenges in evaluating their effects. Spine (Phila Pa 1976) 2008;33:74-80.

4. Department of Health WA. Spinal Pain Model of Care. Perth: Health Networks Branch; 2009.

5. Roberts L, Little P, Chapman J, Cantrell T, Pickering R, Langridge J. The back home trial: general practitioner-supported leaflets may change back pain behavior. Spine (Phila Pa 1976) 2002;27:1821-8.

6. Rosemann T, Joos S, Koerner T, Heiderhoff M, Laux G, Szecsenyi J. Use of a patient information leaflet to influence patient decisions regarding mode of administration of NSAID medications in case of acute low back pain. Eur Spine J 2006;15:1737-41.

7. Evans DW, Breen AC, Pincus T, et al. The effectiveness of a posted information package on the beliefs and behavior of musculoskeletal practitioners: the

UK Chiropractors, Osteopaths, and Musculoskeletal Physiotherapists Low Back Pain ManagemENT (COMPLeMENT) randomized trial. Spine (Phila Pa 1976) 2010;35:858-66.

8. Symonds TL, Burton AK, Tillotson KM, Main CJ. Absence resulting from low back trouble can be reduced by psychosocial intervention at the work place. Spine (Phila Pa 1976) 1995;20:2738-45.

9. Waddell G, Newton M, Henderson I, Somerville D, Main CJ. A Fear-Avoidance Beliefs Questionnaire (FABQ) and the role of fear-avoidance beliefs in chronic low back pain and disability. Pain 1993;52:157-68.

10. Jensen MP, Turner JA, Romano JM, Fisher LD. Comparative reliability and validity of chronic pain intensity measures. Pain 1999;83:157-62.

11. Boonstra AM, Schiphorst Preuper HR, Reneman MF, Posthumus JB, Stewart RE. Reliability and validity of the visual analogue scale for disability in patients with chronic musculoskeletal pain. Int J Rehabil Res 2008;31:165-9.

12. Kamper SJ, Ostelo RWJG, Knol DL, Maher CG, de Vet HCW, Hancock MJ. Global Perceived Effect scales provided reliable assessments of health transition in people with musculoskeletal disorders, but ratings are strongly influenced by current status. J Clin Epidemiol 2010;63:760-6.e1.

13. Briggs AM, Jordan JE, Buchbinder R, et al. Health literacy and beliefs among a community cohort with and without chronic low back pain. Pain 2010;150:275-83.

53144

12345

LOW BACK PAIN IN PRIMARY CARE QUESTIONNAIRE
Time 0 (baseline)

COMPLETION INSTRUCTIONS

Please shade the circles completely ● Please write clearly in the boxes or free text areas 1 2 3 ABCDE

Please read and answer questions carefully. Do not take long to answer the questions, however it is important that you answer every question. There is always a response for your particular situation.

SECTION A: BIOGRAPHICAL INFORMATION

A1. What is your current age?

28 years

A2. Gender:

○ Male ● Female

SECTION B: LOW BACK PAIN

B1. Has your current episode of back pain lasted? (Please shade one only)

● 3 months or more continuously ○ 3 months or more intermittently ○ Less than 3 months

B2. How many days of work or education (eg TAFE, University, re-training scheme) have you missed because of your CURRENT episode of low back pain? (Please shade one only)

○ 0 days ○ 1-2 days ○ 3-7 days ○ 8-14 days ○ 15-30 days
● 1-2 months ○ 2-3 months ○ 3-6 months ○ 6-12 months ○ Over 1 year

B3. Please indicate your highest level of education. (Please shade one only)

○ Never attended school
○ Some primary school
○ Completed primary school
○ Some secondary (high) school
○ Completed secondary (high) school
○ Trade certificate or diploma
● University degree(s)

B4. Considering your low back pain over the last 24 hours, please rate the average severity of low back pain you have experienced on the scale below where 0 means 'no pain' and 10 means 'worst pain imaginable'.

No pain 0	1	2	3	4	5	6	7	8	9	Worst pain 10
○	○	○	○	○	●	○	○	○	○	○

B5. Considering your low back pain over the last 24 hours, please rate how this has affected your normal activities of daily living on the scale below, where 0 means 'not affected at all' and 10 means 'unable to perform any activities of daily living'.

Not affected at all 0	1	2	3	4	5	6	7	8	9	Unable to perform any activities of daily living 10
○	○	○	○	○	○	●	○	○	○	○

53144

12345

B6. For each statement below please indicate how much physical activities (such as bending, lifting, walking or driving) and work activities affect or would affect your back pain. (Please shade one circle for each question)

	Completely disagree 0	Moderately disagree 1	Slightly disagree 2	Unsure 3	Slightly agree 4	Moderately agree 5	Completely agree 6
a) My pain was caused by physical activity.	●	○	○	○	○	○	○
b) Physical activity makes my pain worse.	○	●	○	○	○	○	○
c) Physical activity might harm my back.	○	○	●	○	○	○	○
d) I should not do physical activities which (might) make my pain worse.	○	○	○	●	○	○	○
e) I cannot do physical activities which (might) make my pain worse.	○	○	○	○	●	○	○
f) My pain was caused by my work or by an accident at work.	○	○	○	○	○	●	○
g) My work aggravated my pain.	○	○	○	○	○	○	●
h) I have a claim for compensation for my pain.	○	○	○	○	○	●	○
i) My work is too heavy for me.	○	○	○	○	●	○	○
j) My work makes or would make my pain worse.	○	○	○	●	○	○	○
k) My work might harm my back.	○	○	●	○	○	○	○
l) I should not do my normal work with my present pain.	○	●	○	○	○	○	○
m) I cannot do my normal work with my present pain.	●	○	○	○	○	○	○
n) I cannot do my normal work until my pain is treated.	○	●	○	○	○	○	○
o) I do not think that I will be back to my normal work within 3 months.	○	○	●	○	○	○	○
p) *I do not think that I will ever be able to go back to my normal work.*	○	○	○	●	○	○	○

B7. We are interested in what you think about back pain generally. Please indicate your general views about back trouble. (Please answer the statements below by selecting one response to each statement)

	Completely disagree 1	2	3	4	Completely agree 5
a) There is no real treatment for back trouble.	●	○	○	○	○
b) Back trouble will eventually stop you from working.	○	●	○	○	○
c) Back trouble means periods of pain for the rest of one's life.	○	○	●	○	○
d) Doctors cannot do anything for back trouble.	○	○	○	●	○
e) A bad back should be exercised.	○	○	○	○	●
f) Back trouble makes everything in life worse.	○	○	○	●	○
g) Surgery is the *most effective* way to treat back trouble.	○	○	●	○	○
h) Back trouble may mean you end up in a wheelchair.	○	●	○	○	○
i) Alternative treatments are the answer to back trouble.	●	○	○	○	○
j) Back trouble means long periods of time off work.	○	●	○	○	○
k) Medication is the *only way* of relieving back trouble.	○	○	●	○	○
l) Once you have had back trouble there is *always* a weakness.	○	○	○	●	○
m) Back trouble *must* be rested.	○	○	○	○	●
n) Later in life back trouble gets progressively worse.	○	○	○	●	○

LOW BACK PAIN IN PRIMARY CARE QUESTIONNAIRE
Time 1 (at 2 weeks post initial questionnaire)

COMPLETION INSTRUCTIONS

Please shade the circles completely ● Please write clearly in the boxes or free text areas | 1 | 2 | 3 | ABCDE |

Please read and answer questions carefully. Do not take long to answer the questions, however it is important that you answer every question. There is always a response for your particular situation.

SECTION A: LOW BACK PAIN

A1. Considering your low back pain over the last 24 hours, please rate the average severity of low back pain you have experienced on the scale below where 0 means 'no pain' and 10 means 'worst pain imaginable'.

No pain 0	1	2	3	4	5	6	7	8	9	Worst pain 10
○	○	○	○	○	○	○	○	○	○	○

A2. Considering your low back pain over the last 24 hours, please rate how this has affected your normal activities of daily living on the scale below, where 0 means 'not affected at all' and 10 means 'unable to perform any activities of daily living'.

Not affected at all 0	1	2	3	4	5	6	7	8	9	Unable to perform any activities of daily living 10
○	○	○	○	○	○	○	○	○	○	○

A3. For each statement below please indicate how much physical activities (such as bending, lifting, walking or driving) and work activities affect or would affect your back pain. (Please shade one circle for each question)

	Completely disagree 0	Moderately disagree 1	Slightly disagree 2	Unsure 3	Slightly agree 4	Moderately agree 5	Completely agree 6
a) My pain was caused by physical activity.	○	○	○	○	○	○	○
b) Physical activity makes my pain worse.	○	○	○	○	○	○	○
c) Physical activity might harm my back.	○	○	○	○	○	○	○
d) I should not do physical activities which (might) make my pain worse.	○	○	○	○	○	○	○
e) I cannot do physical activities which (might) make my pain worse.	○	○	○	○	○	○	○
f) My pain was caused by my work or by an accident at work.	○	○	○	○	○	○	○
g) My work aggravated my pain.	○	○	○	○	○	○	○
h) I have a claim for compensation for my pain.	○	○	○	○	○	○	○
i) My work is too heavy for me.	○	○	○	○	○	○	○
j) My work makes or would make my pain worse.	○	○	○	○	○	○	○
k) My work might harm my back.	○	○	○	○	○	○	○

A3. (continued) For each statement below please indicate how much physical activities (such as bending, lifting, walking or driving) and work activities affect or would affect your back pain.
(Please shade **one** circle for **each** question)

	Completely disagree 0	Moderately disagree 1	Slightly disagree 2	Unsure 3	Slightly agree 4	Moderately agree 5	Completely agree 6
l) I should not do my normal work with my present pain.	○	○	○	○	○	○	○
m) I cannot do my normal work with my present pain.	○	○	○	○	○	○	○
n) I cannot do my normal work until my pain is treated.	○	○	○	○	○	○	○
o) I do not think that I will be back to my normal work within 3 months.	○	○	○	○	○	○	○
p) I do not think that I will ever be able to go back to my normal work.	○	○	○	○	○	○	○

A4. We are interested in what you think about back pain generally. Please indicate your general views about back trouble.
(Please answer the statements below by selecting **one** response to **each** statement)

	Completely disagree 1	2	3	4	Completely agree 5
a) There is no real treatment for back trouble.	○	○	○	○	○
b) Back trouble will eventually stop you from working.	○	○	○	○	○
c) Back trouble means periods of pain for the rest of one's life.	○	○	○	○	○
d) Doctors cannot do anything for back trouble.	○	○	○	○	○
e) A bad back should be exercised.	○	○	○	○	○
f) Back trouble makes everything in life worse.	○	○	○	○	○
g) Surgery is the *most effective* way to treat back trouble.	○	○	○	○	○
h) Back trouble may mean you end up in a wheelchair.	○	○	○	○	○
i) Alternative treatments are the answer to back trouble.	○	○	○	○	○
j) Back trouble means long periods of time off work.	○	○	○	○	○
k) Medication is the *only way* of relieving back trouble.	○	○	○	○	○
l) Once you have had back trouble there is *always* a weakness.	○	○	○	○	○
m) Back trouble *must* be rested.	○	○	○	○	○
n) Later in life back trouble gets progressively worse.	○	○	○	○	○

SECTION B: THE PAMPHLET

B1. Please indicate on the scale below how useful you found the "Consumer's Guide to Managing Back Pain" pamphlet, where 0 means 'not at all useful' and 10 means 'extremely useful'.

This question only applies to those people who received a copy of the pamphlet.

Not at all useful 0	1	2	3	4	5	6	7	8	9	Extremely useful 10	Not applicable
○	○	○	○	○	○	○	○	○	○	○	○

35788

LOW BACK PAIN IN PRIMARY CARE QUESTIONNAIRE
Time 2 (at 8 weeks post initial questionnaire)

COMPLETION INSTRUCTIONS

Please shade the circles completely ●   Please write clearly in the boxes or free text areas | 1 | 2 | 3 | **ABCDE**

Please read and answer questions carefully. Do not take long to answer the questions, however it is important that you answer every question. There is always a response for your particular situation.

SECTION A: LOW BACK PAIN

A1. Considering your low back pain over the last 24 hours, please rate the <u>average severity</u> of low back pain you have experienced on the scale below where 0 means 'no pain' and 10 means 'worst pain imaginable'.

No pain 0	1	2	3	4	5	6	7	8	9	Worst pain 10
○	○	○	○	○	○	○	○	○	○	○

A2. Considering your low back pain over the last 24 hours, please rate how this has affected your normal activities of daily living on the scale below, where 0 means '<u>not affected at all</u>' and 10 means '<u>unable to perform any activities of daily living</u>'.

Not affected at all 0	1	2	3	4	5	6	7	8	9	Unable to perform any activities of daily living 10
○	○	○	○	○	○	○	○	○	○	○

A3. For each statement below please indicate how much physical activities (such as bending, lifting, walking or driving) and work activities affect or would affect your back pain. (Please shade <u>one</u> circle for <u>each</u> question)

	Completely disagree 0	Moderately disagree 1	Slightly disagree 2	Unsure 3	Slightly agree 4	Moderately agree 5	Completely agree 6
a) My pain was caused by physical activity.	○	○	○	○	○	○	○
b) Physical activity makes my pain worse.	○	○	○	○	○	○	○
c) Physical activity might harm my back.	○	○	○	○	○	○	○
d) I should not do physical activities which (might) make my pain worse.	○	○	○	○	○	○	○
e) I cannot do physical activities which (might) make my pain worse.	○	○	○	○	○	○	○
f) My pain was caused by my work or by an accident at work.	○	○	○	○	○	○	○
g) My work aggravated my pain.	○	○	○	○	○	○	○
h) I have a claim for compensation for my pain.	○	○	○	○	○	○	○
i) My work is too heavy for me.	○	○	○	○	○	○	○
j) My work makes or would make my pain worse.	○	○	○	○	○	○	○
k) My work might harm my back.	○	○	○	○	○	○	○

A3. (continued) For each statement below please indicate how much physical activities (such as bending, lifting, walking or driving) and work activities affect or would affect your back pain.
(Please shade **one** circle for **each** question)

	Completely disagree 0	Moderately disagree 1	Slightly disagree 2	Unsure 3	Slightly agree 4	Moderately agree 5	Completely agree 6
l) I should not do my normal work with my present pain.	○	○	○	○	○	○	○
m) I cannot do my normal work with my present pain.	○	○	○	○	○	○	○
n) I cannot do my normal work until my pain is treated.	○	○	○	○	○	○	○
o) I do not think that I will be back to my normal work within 3 months.	○	○	○	○	○	○	○
p) I do not think that I will ever be able to go back to my normal work.	○	○	○	○	○	○	○

A4. We are interested in what you think about back pain generally. Please indicate your general views about back trouble.
(Please answer the statements below by selecting **one** response to **each** statement)

	Completely disagree 1	2	3	4	Completely agree 5
a) There is no real treatment for back trouble.	○	○	○	○	○
b) Back trouble will eventually stop you from working.	○	○	○	○	○
c) Back trouble means periods of pain for the rest of one's life.	○	○	○	○	○
d) Doctors cannot do anything for back trouble.	○	○	○	○	○
e) A bad back should be exercised.	○	○	○	○	○
f) Back trouble makes everything in life worse.	○	○	○	○	○
g) Surgery is the *most effective* way to treat back trouble.	○	○	○	○	○
h) Back trouble may mean you end up in a wheelchair.	○	○	○	○	○
i) Alternative treatments are the answer to back trouble.	○	○	○	○	○
j) Back trouble means long periods of time off work.	○	○	○	○	○
k) Medication is the *only way* of relieving back trouble.	○	○	○	○	○
l) Once you have had back trouble there is *always* a weakness.	○	○	○	○	○
m) Back trouble *must* be rested.	○	○	○	○	○
n) Later in life back trouble gets progressively worse.	○	○	○	○	○

SECTION B: THE PAMPHLET

B1. Please indicate on the scale below how useful you found the "Consumer's Guide to Managing Back Pain" pamphlet, where 0 means '<u>not at all useful</u>' and 10 means '<u>extremely useful</u>'.

This question only applies to those people who received a copy of the pamphlet.

Not at all useful 0	1	2	3	4	5	6	7	8	9	Extremely useful 10	Not applicable
○	○	○	○	○	○	○	○	○	○	○	○

SAVANT SURVEYS & STRATEGIES

This questionnaire was constructed in a scannable format by
SAVANT Surveys and Strategies
www.savant.net.au
(08) 9325 1500
